# Maintenance of Type IV Secretion Function During Helicobacter pylori Infection in Mice

**DOI:** 10.1128/mBio.03147-20

**Published:** 2020-12-22

**Authors:** Emma C. Skoog, Miriam E. Martin, Roberto M. Barrozo, Lori M. Hansen, Lucy P. Cai, Seung-Joo Lee, Joseph M. Benoun, Stephen J. McSorley, Jay V. Solnick

**Affiliations:** a Department of Medicine, University of California, Davis, Davis, California, USA; b Department of Microbiology & Immunology, University of California, Davis, Davis, California, USA; c Department of Anatomy, Physiology, and Cell Biology, University of California, Davis, Davis, California, USA; d Department of Microbiology and Molecular Genetics, University of California, Davis, Davis, California, USA; e Center for Immunology and Infectious Diseases, University of California, Davis, Davis, California, USA; f Genentech, South San Francisco, California, USA; g Alector, South San Francisco, California, USA; h Kite Pharma, Santa Monica, California, USA; Rutgers University

**Keywords:** *Helicobacter pylori*, *Salmonella*, type IV secretion system, *cagY*, pathogenicity island

## Abstract

The Helicobacter pylori type IV secretion system (T4SS) encoded on the *cag* pathogenicity island (*cag*PAI) secretes the CagA oncoprotein and other effectors into the gastric epithelium. During murine infection, T4SS function is lost in an immune-dependent manner, typically as a result of in-frame recombination in the middle repeat region of *cagY*, though single nucleotide polymorphisms (SNPs) in *cagY* or in other essential genes may also occur. Loss of T4SS function also occurs in gerbils, nonhuman primates, and humans, suggesting that it is biologically relevant and not simply an artifact of the murine model. Here, we sought to identify physiologically relevant conditions under which T4SS function is maintained in the murine model. We found that loss of H. pylori T4SS function in mice was blunted by systemic *Salmonella* coinfection and completely eliminated by dietary iron restriction. Both have epidemiologic parallels in humans, since H. pylori strains from individuals in developing countries, where iron deficiency and systemic infections are common, are also more often *cag*PAI^+^ than strains from developed countries. These results have implications for our fundamental understanding of the *cag*PAI and also provide experimental tools that permit the study of T4SS function in the murine model.

## INTRODUCTION

Infection with Helicobacter pylori causes chronic gastric inflammation that sometimes progresses to peptic ulcer disease or gastric adenocarcinoma, which is the third most common cause of cancer mortality worldwide (1). The virulence locus most strongly associated with disease is the type IV secretion system (T4SS) encoded on the cytotoxin-associated gene pathogenicity island (*cag*PAI). The H. pylori T4SS is essential for injection of several known bacterial effectors into host cells, including the CagA oncoprotein (2, 3), chromosomal DNA (4), peptidoglycan (5), and ADP-heptose, an intermediate metabolite of lipopolysaccharide biosynthesis (6–8). T4SS-dependent translocation of effectors activates the NF-κB inflammatory pathway and induces interleukin 8 (IL-8), a chemokine that recruits neutrophils to the site of infection and promotes chronic inflammation (9, 10).

Experimental H. pylori infection in mouse models usually results in loss of T4SS function, typically measured by the capacity of the recovered strains to induce IL-8 or translocate CagA (11). We recently found that this is most often due to recombination in *cagY*, an ortholog of virB10 that encodes an essential protein found in all known T4SSs (12), though changes in other essential *cag*PAI genes also occur commonly (13). Recombination in *cagY* occurs in what has been called the middle repeat region (MRR)—a segment of *cagY* that has an extraordinary number of directs repeats (14)—leaving the open reading frame intact and the protein expressed but altering T4SS function (12). The DNA repeats encode a series of amino acid motifs, typically consisting of 30 to 40 residues (15), one or more of which is lost (or sometimes gained) by recombination events. This yields a potentially large number of variant *cagY* alleles, some functional and some not, though to date, it has not been possible to distinguish them based simply on sequence. Loss of T4SS function is driven by the host immune response (16) and is dependent on CD4^+^ T cells and interferon gamma (IFN-γ). The precise mechanism by which alterations in the MRR motif structure regulate T4SS function is unclear. However, the MRR is expressed on the bacterial surface, and motifs that confer function also enable the bacterial cell to bind β1 integrin (17). Since β1 integrin was previously shown to be essential for T4SS function (18, 19), these results suggested that alteration in CagY binding to β1 integrin might mediate changes in T4SS function. However, this conclusion remains speculative in view of recent studies showing that carcinoembryonic antigen-related cell adhesion molecule (CEACAM) receptors but not integrins are essential for CagA translocation (20).

CagY-mediated loss of T4SS function has been observed not only in mice but also in rhesus macaques (12) and in gerbils (21), though some gerbil-adapted strains have retained function (22). Therefore, it seems likely that loss of T4SS by changes in CagY is not simply an artifact observed in animal models but rather reflects an aspect of H. pylori biology that is also critical for chronic human infection. This is also supported by the fact that all known *cagY* sequences contain an MRR and that isogenic H. pylori strains with *cagY*-dependent differences in T4SS function have been recovered from chronically infected humans (16). Since the T4SS enhances gastritis and reduces bacterial load (23, 24), loss of T4SS function from the bacterial perspective may serve to reduce inflammation so as to maintain adequate colonization and transmission to a new host. Yet, most *cag*PAI^+^ H. pylori strains recovered from humans (25) and from naturally infected rhesus monkeys (26) have a functional T4SS, and so this is apparently the homeostatic state. But there must be circumstances in natural human infection during which loss of T4SS function is selected. One possibility is that concurrent systemic infection with another pathogen may induce inflammatory cytokines, which suppress H. pylori bacterial load in a non-antigen-specific manner, and selects for strains that can overcome this immune pressure by loss of T4SS function. Since the *cag*PAI is important for iron acquisition (27, 28), another possibility is that PAI function is maintained under iron-limiting conditions but not when iron is replete. Here, we address these hypotheses in the H. pylori mouse model by examining the effects of *Salmonella* coinfection and iron deprivation on PAI function. The results demonstrate that T4SS function is actually maintained by systemic coinfection with *Salmonella* and also by iron deprivation, both of which are relevant to natural human infection, particularly in developing countries where H. pylori is most common.

## RESULTS

### Characterization of the H. pylori-*Salmonella* coinfection model.

**FIG 1:**
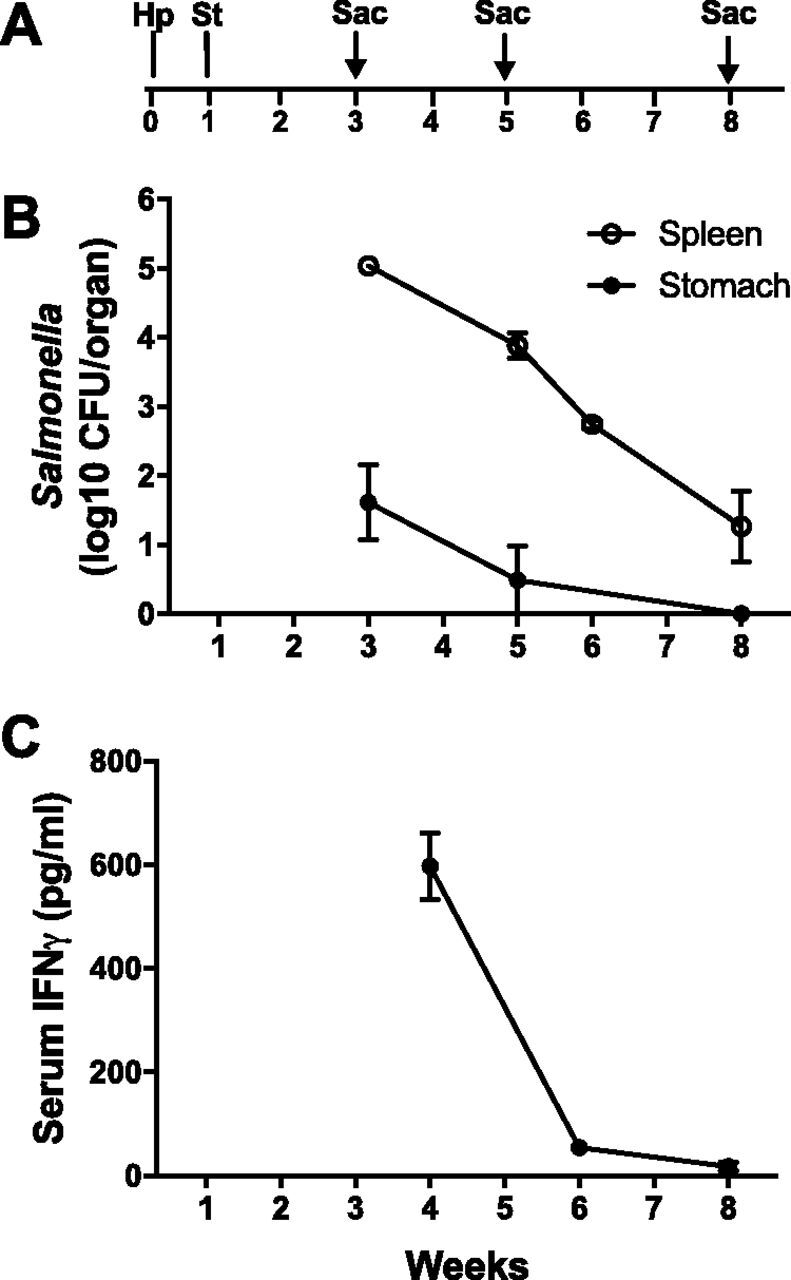
Characterization of the H. pylori-*Salmonella* coinfection model. (A) Schematic time frame of the H. pylori-*Salmonella* coinfection model. Mice were orally gavaged with H. pylori PMSS1 (Hp), infected intravenously with *Salmonella* Typhimurium (St) 1 week later, and sacrificed (Sac) 3, 5, or 8 weeks after H. pylori infection. (B) There was robust colonization of the spleen with *Salmonella*, which decreased over the course of infection. *Salmonella* was also present initially in the stomach at much lower quantities but was undetectable by 8 weeks. (C) Serum IFN-γ levels were high 3 weeks after *Salmonella* infection and declined rapidly. Data represent the means ± SEMs from 4 to 8 mice at each time point.

The Salmonella enterica serovar Typhimurium challenge model with live-attenuated strain BRD509 was previously described (29). Intravenous inoculation is followed rapidly by high bacterial burden in the spleen and expansion of IFN-γ CD4^+^ T cells, which peak 1 to 2 weeks later and largely dissipate by 6 to 8 weeks. C57BL/6 mice were orally gavaged with H. pylori PMSS1, injected with *S.* Typhimurium BRD509 intravenously (i.v.) 1 week later, and then sacrificed 3, 5, or 8 weeks postinoculation (p.i.) with H. pylori (Fig. 1A). As expected, *S.* Typhimurium challenge produced a robust systemic infection, with rapid colonization of the spleen that decreased over the course of the 8-week experiment (Fig. 1B). *Salmonella* was also recovered from gastric tissue, though in much smaller numbers. Serum IFN-γ detected by enzyme-linked immunosorbent assay (ELISA) was markedly elevated 3 weeks after *Salmonella* challenge in mice infected with H. pylori and declined rapidly (Fig. 1C). Eight weeks p.i., when H. pylori colonization and T4SS were characterized, IFN-γ levels were low, and mice had largely recovered from infection with *Salmonella*, which was no longer detectable in the stomach.

### *Salmonella* coinfection decreases H. pylori colonization and enhances local and systemic inflammation.

**FIG 2:**
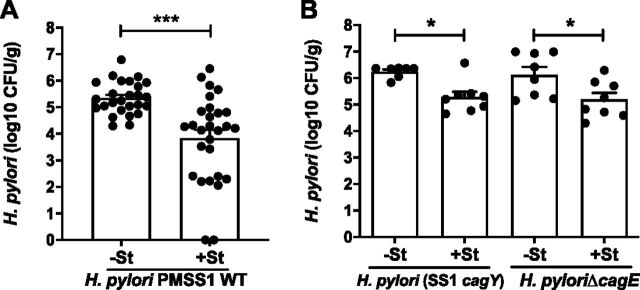
*Salmonella* coinfection decreases H. pylori colonization. (A) Colonization of WT H. pylori in the stomach was decreased 8 weeks p.i. in *Salmonella*-coinfected animals (+St) compared to that in animals with H. pylori infection alone (−St). (B) Colonization with H. pylori SS1*cagY* (PMSS1 with *cagY* from SS1) and H. pylori Δ*cagE*, which have defective T4SSs, was also decreased when mice were coinfected with *Salmonella*. Each data point represents one mouse. Bars indicate means ± SEMs. *, *P* < 0.05; ***, *P* < 0.005.

**FIG 3:**
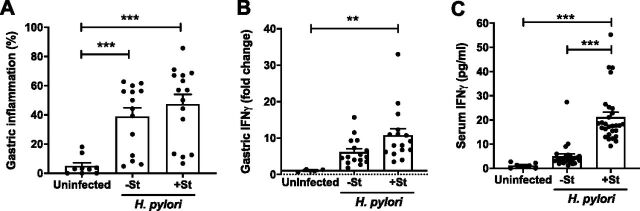
*Salmonella* coinfection enhances local and systemic inflammation. Gastric inflammation (mean percent fields containing neutrophils, mononuclear cells, or metaplasia) (A) and IFN-γ (B) were induced by H. pylori infection alone and increased slightly in coinfections with *Salmonella*. (C)Infection with *Salmonella* markedly increased serum IFN-γ compared to that with H. pylori alone or in uninfected mice. Data are from mice 8 weeks after H. pylori infection or at an equivalent age for uninfected mice. Each data point represents one mouse. Bars indicate means ± SEMs. **, *P* < 0.01; ***, *P* < 0.005.

H. pylori colonization was assessed 8 weeks p.i. (7 weeks after *Salmonella* challenge), when *cagY* recombination and loss of T4SS function become apparent. Mice challenged with wild-type (WT) H. pylori harbored 10^5^ CFU/g of gastric tissue, which was approximately 10-fold lower in mice that also received *S.* Typhimurium (Fig. 2A). Colonization with H. pylori SS1*cagY* or H. pylori Δ*cagE*, which have a nonfunctional T4SS, was greater than with WT H. pylori but showed a similar decrease when coinfected with *Salmonella* (Fig. 2B). Gastric inflammation was induced by H. pylori infection and was somewhat increased in mice coinfected with *S.* Typhimurium (Fig. 3A). Since IFN-γ is induced by *Salmonella* challenge (Fig. 1) and is important for control of H. pylori (16), we also examined IFN-γ levels in serum and gastric tissue 8 weeks p.i. The results showed that *Salmonella* coinfection increased IFN-γ expression in gastric tissue (Fig. 3B) and in serum (Fig. 3C) compared to that with H. pylori alone, though only the latter was statistically significant. Together, these results show that coinfection with *Salmonella* induces a systemic and local inflammatory response that is associated with reduced H. pylori colonization, independent of T4SS function.

### Gastric overexpression of IFN-γ is sufficient to decrease H. pylori colonization.

**FIG 4:**
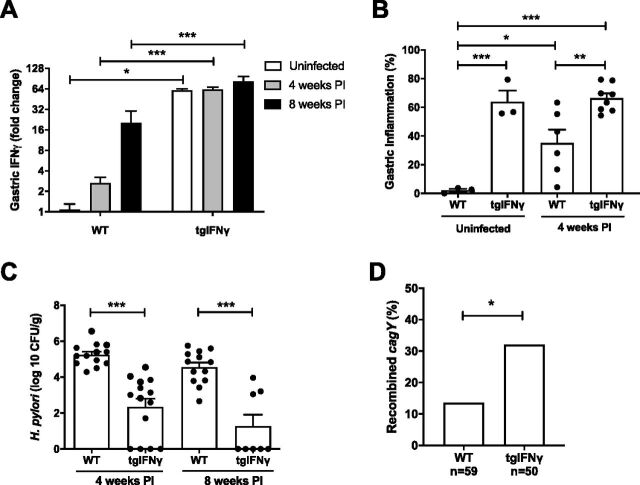
Gastric overexpression of IFN-γ inhibits H. pylori colonization. (A) Gastric IFN-γ expression was greater in heterozygous tgIFN-γ mice than in WT mice and tended to increase further by H. pylori infection, particularly in WT mice. (B) Gastric inflammation was increased by overexpression of IFN-γ and by H. pylori infection in WT but not in the highly inflamed transgenic mice. (C) H. pylori colonization was reduced at 4 weeks p.i. and mostly cleared after 8 weeks in transgenic mice. (D) Colonies (3 to 6 per mouse) isolated from tgIFN-γ mice 4 weeks p.i. were more frequently found to have recombined *cagY* than colonies from WT mice. Bars indicate means ± SEMs. *, *P* < 0.05; **P < 0.01; ***, *P* < 0.005.

To further characterize the role of IFN-γ in controlling H. pylori colonization, we challenged heterozygous mice overexpressing mouse IFN-γ under the control of the stomach-specific H^+^/K^+^ ATPase β promoter (tgIFN-γ). Gastric IFN-γ transcript levels were increased by H. pylori infection and were markedly greater in tgIFN-γ than in WT mice (Fig. 4A). Gastric inflammation was also increased in tgIFN-γ compared to that in WT mice, both uninfected and 4 weeks p.i. (Fig. 4B), and was accompanied by decreased H. pylori bacterial load at 4 and at 8 weeks p.i., when most animals had cleared the infection (Fig. 4C). Functional T cells are required to control H. pylori infection (16), and they are also the major source of IFN-γ. To determine if IFN-γ is sufficient to reduce H. pylori colonization in the absence of T cells, we compared infection in TCR^−/−^ mice and TCR^−/−^ mice bearing the IFN-γ transgene. As in mice with functional T cells (Fig. 4A), IFN-γ expression was increased in T cell receptor-deficient (TCR^−/−^) mice expressing the IFN-γ transgene (see Fig. S1A in the supplemental material), while H. pylori colonization was decreased (Fig. S1B). Similar to previous results (16), *cagY* recombination was eliminated in TCR^−/−^ mice but increased somewhat by overexpression of IFN-γ, even in the absence of functional T cells (Fig. S1C). These results suggest that reduction in H. pylori colonization in the *Salmonella* coinfection model may be explained, at least in part, by increases in IFN-γ-mediated inflammation.

10.1128/mBio.03147-20.1 FIG S1 Transgenic expression of IFN-γ inhibits H. pylori colonization independent of T cells. (A) Gastric IFN-γ expression was increased in H. pylori-uninfected and -infected TCR^−/−^ mice harboring the IFN-γ transgene. (B) Transgenic IFN-γ expression decreased H. pylori colonization 8 weeks p.i. in TCR^−/−^ mice. (C) *cagY* recombination occurred in colonies from WT and TCR^−/−^ mice harboring the IFN-γ transgene but not in colonies isolated from TCR^−/−^ mice. Bars indicate means ± SEMs. *, *P* < 0.05; **, *P < *0.01; ***, *P* < 0.005. Download FIG S1, PDF file, 0.1 MB. Copyright © 2020 Skoog et al. 2020 Skoog et al. This content is distributed under the terms of the Creative Commons Attribution 4.0 International license.

### *Salmonella* coinfection promotes retention of H. pylori T4SS function.

**FIG 5:**
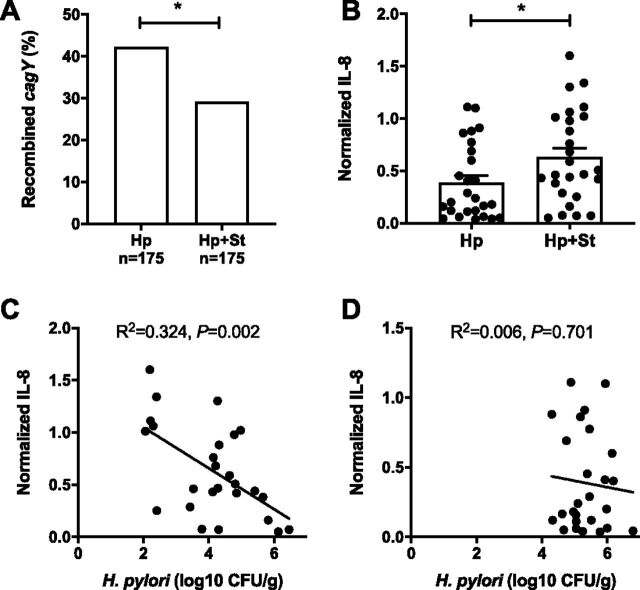
*Salmonella* coinfection enhances H. pylori T4SS function. (A) Fewer H. pylori colonies (3 to 6 per mouse) from coinfected mice showed recombination of *cagY* by RFLP analysis (Fisher’s exact test, *P* < 0.05). (B) T4SS function (IL-8 induction) was greater for H. pylori isolated from *Salmonella*-coinfected mice than from mice infected with H. pylori alone. Each data point represents a sweep of H. pylori colonies isolated from one mouse. In *Salmonella*-coinfected mice, the colonization level of H. pylori correlated inversely with the capacity to induce IL-8 in AGS cells (C), while this was not the case with H. pylori alone (D). Bars indicate means ± SEMs. *, *P* < 0.05.

We previously found that increased gastritis in IL-10^−/−^ mice is associated with decreased H. pylori colonization (16), similar to our findings here in tgIFN-γ mice. However, some IL-10^−/−^ mice were colonized at levels similar to those of WT mice, and H. pylori recovered from them had typically recombined *cagY*, suggesting that loss of T4SS function permits increased colonization in the face of a robust immune response. Consistent with this observation, we also found that overexpression of IFN-γ caused increased *cagY* recombination (Fig. 4D), even in the absence of functional T cells (Fig. S1C). Since *Salmonella* coinfection enhances the systemic and local inflammatory responses and decreases H. pylori colonization, we hypothesized that it too might select for *cagY*-mediated loss of T4SS function and enable H. pylori to partially escape increased immune pressure from IFN-γ and other inflammatory cytokines. However, we observed the opposite result. *cagY* recombination was reduced (Fig. 5A) and T4SS function was increased (Fig. 5B) in H. pylori colonies recovered from coinfected mice compared to those in mice infected with H. pylori alone. H. pylori colonization was inversely correlated with T4SS function in coinfected mice (Fig. 5C) but not in mice infected with H. pylori alone, where colonization was overall greater and less variable (Fig. 5D).

### H. pylori T4SS function is retained in iron-deficient mice.

*In vitro* experiments suggest that CagA translocation via the T4SS serves to increase iron acquisition from host cells (28). These results are consistent with *in vivo* studies demonstrating that deletion of *cagA* impairs H. pylori colonization in iron-deficient, but not iron-replete, gerbils (28). Moreover, H. pylori strains recovered from iron-deficient gerbils, or grown *in vitro* under iron-deficient conditions, demonstrate greater numbers of T4SS pili and show enhanced T4SS function (30). Since *Salmonella* induces anemia (31) and inflammation-mediated iron sequestration (32), we hypothesized that *Salmonella* coinfection might select for a functional T4SS by competing with H. pylori for iron. To first test the effects of anemia on colonization and T4SS function, we gavaged H. pylori into EPO^−/−^ mice bearing a homozygous disruption in the 5′ untranslated region of the erythropoietin gene (Epo-Tag^h^), which reduces whole-body erythropoietin expression (33). EPO^−/−^ mice are severely anemic, with a hematocrit level approximately half that of WT mice (see Fig. S2A), though they may not be iron deficient. Hematocrit was further reduced by H. pylori infection in both WT and EPO^−/−^ mice, but H. pylori colonization and T4SS function were unaffected (Fig. S2B and C).

10.1128/mBio.03147-20.2 FIG S2 Erythropoietin deficiency in mice causes anemia but does not affect H. pylori colonization or T4SS function. (A) EPO^−/−^ mice have markedly reduced hematocrit levels, which are further reduced 8 weeks after H. pylori infection. H. pylori colonization (B) and T4SS function (C) are the same in WT and EPO^−/−^ mice. Bars indicate means ± SEMs. ns, not significant; **, *P* < 0.01; ***, *P* < 0.005. Download FIG S2, PDF file, 0.1 MB. Copyright © 2020 Skoog et al. 2020 Skoog et al. This content is distributed under the terms of the Creative Commons Attribution 4.0 International license.

**FIG 6:**
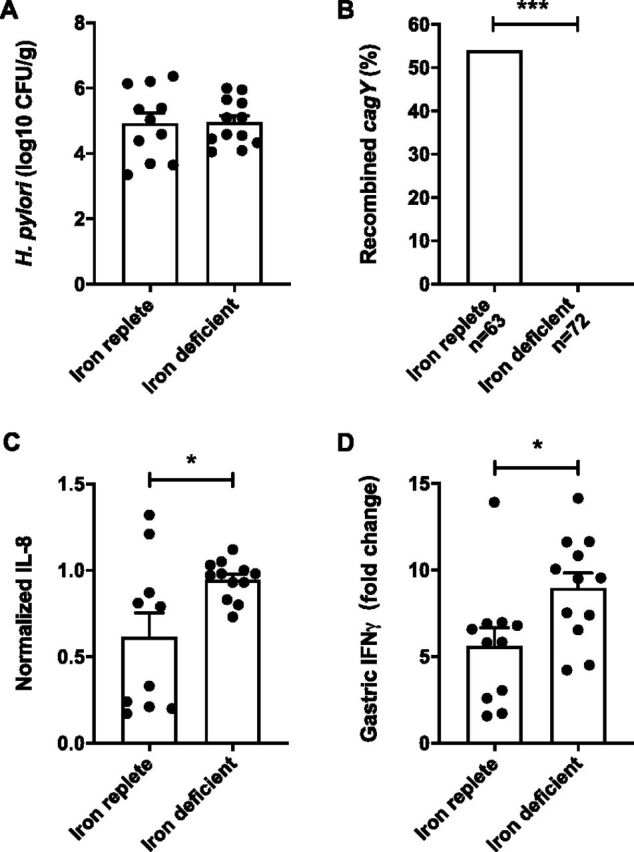
H. pylori *cagY* and T4SS function are maintained in iron-deficient mice. (A) At 8 weeks p.i., the colonization load of H. pylori was not affected by the iron-deficient diet. However, compared to colonies (3 to 6/mouse) from iron-replete mice, H. pylori colonies from mice fed an iron-deficient diet maintained T4SS function (induction of IL-8) indistinguishable from that WT PMSS1 (B), and showed no recombination of *cagY* by RFLP analysis (C) (Fisher’s exact test, *P* < 0.0001). (D) Gastric IFN-γ expression was greater in iron-deficient mice that were colonized with H. pylori with a fully functional T4SS than in iron-replete mice. Bars indicate means ± SEMs. *, *P* < 0.05; ***, *P* < 0.0001.

Anemia can occur with or without iron deficiency. To test specifically for the role of iron deficiency on H. pylori colonization and T4SS function, we compared H. pylori infections in mice fed for 5 weeks with an iron-deficient diet (2 to 6 ppm iron) to infections in mice fed a standard diet (200 ppm iron). H. pylori infection was followed by a decrease in food intake, which was restored in mice fed an iron-replete diet but not in those fed an iron-deficient diet (see Fig. S3A). The iron-deficient mice also weighed slightly less than control mice at the end of the experiment (Fig. S3B). Serum iron levels were highly variable in mice fed an iron-deficient diet, though anemia was severe and uniform (Fig. S3C and D). Similar to anemia in EPO^−/−^ mice, iron deficiency anemia did not affect H. pylori colonization (Fig. 6A). However, the effect on T4SS function was dramatic, with no *cagY* recombination and no loss of IL-8 induction in iron-deficient mice 8 weeks p.i. (Fig. 6B and C). Gastric IFN-γ levels were also increased in H. pylori-infected iron-deficient mice, likely as a result of the increased T4SS activity (Fig. 6D).

10.1128/mBio.03147-20.3 FIG S3 Iron deficient diet causes anemia. (A) Three-week-old mice were fed for 5 weeks an iron-depleted or iron-replete diet and then infected with H. pylori. H. pylori infection was followed by a sudden drop in food intake, which recovered in mice fed an iron-replete diet but not in those fed an iron-depleted diet. (B) Consistent with lower food intake, iron-deficient mice weighed less than iron-replete mice 8 weeks after H. pylori infection. Serum iron levels 8 weeks p.i. were variable in mice fed an iron-deficient diet (C), while hematocrit was uniformly and substantially lower than in mice fed an iron-replete diet (D). Bars indicate means ± SEMs. *, *P* < 0.05; ***, *P* < 0.005. Download FIG S3, PDF file, 0.1 MB. Copyright © 2020 Skoog et al. 2020 Skoog et al. This content is distributed under the terms of the Creative Commons Attribution 4.0 International license.

### Role of iron deficiency in *Salmonella* coinfection.

**FIG 7:**
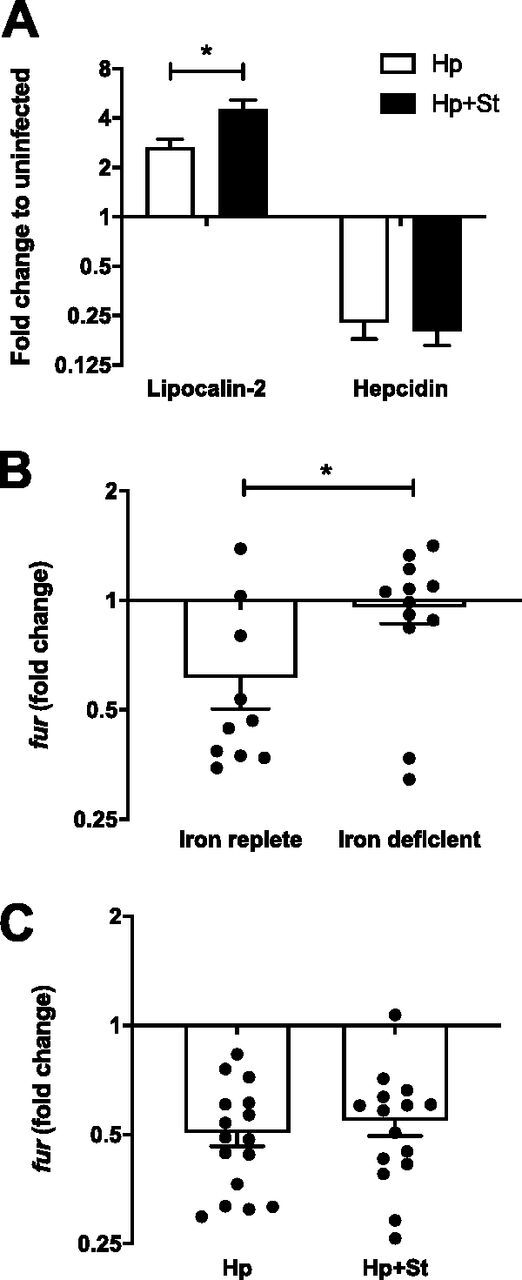
*Salmonella* coinfection does not limit H. pylori iron availability. (A) Gastric expression of lipocalin-2 was greater in H. pylori- and *Salmonella*-coinfected mice than in those infected with H. pylori alone. Hepcidin expression was decreased by H. pylori infection with or without *Salmonella* coinfection. (B) Fold change expression of *fur* relative to input PMSS1 was higher in low-passage-number H. pylori isolated from mice fed an iron-deficient than in iron-replete controls. (C) Fold change expression of *fur* relative to input PMSS1 from H. pylori isolated 8 weeks p.i. and cocultured with AGS cells was not affected by *Salmonella* coinfection. Bars indicate means ± SEMs. *, *P* < 0.05.

Since iron deficiency maintains T4SS function in mice, we hypothesized that retention of T4SS function during *Salmonella* coinfection may be a result of iron starvation. To determine if *Salmonella* coinfection induces iron deficiency, we analyzed gastric expression of lipocalin-2, hepcidin, and fur, which are known to be regulated by iron and therefore serve as a functional readout of iron deficiency. *Salmonella* induces lipocalin-2 in the gut, which binds siderophores from *Enterobacteriaceae* and enables *Salmonella* to overcome colonization resistance (32). Although *Salmonella* coinfection increased gastric lipocalin-2 expression somewhat more than H. pylori alone (Fig. 7A), it seems unlikely that this is relevant for iron sequestration from H. pylori in the stomach, since H. pylori is not known to produce siderophores. Hepcidin is also upregulated in *Salmonella* infection, causing iron retention in macrophages (34). We confirmed previous observations (35) that H. pylori decreases hepcidin expression in mice, but this was unaffected by *Salmonella* coinfection (Fig. 7A). Finally, we investigated expression of the H. pylori ferric uptake regulator, *fur*, which is activated by iron restriction (36). Since we could not readily detect low levels of H. pylori gene expression from infected gastric tissue, we examined *fur* expression in H. pylori cocultured with AGS cells to mimic interactions with the gastric epithelia. Low-passage-number H. pylori isolated from iron-deficient mice showed greater expression of *fur* than H. pylori from iron-replete mice, which validated that the assay served as a bacterial readout of iron deprivation (Fig. 7B). However, *fur* expression in H. pylori was unaffected by coinfection with *Salmonella* (Fig. 7C). Together, these results suggest that neither host nor bacterial expression support the hypothesis that *Salmonella* coinfection in mice limits iron availability for H. pylori.

## DISCUSSION

After the initial demonstration in mice (11), loss of T4SS function has been demonstrated in gerbils (21), rhesus macaques (12), and humans (16, 37), suggesting that modulation of T4SS function is an important feature of the biology of H. pylori. The mechanism is typically in-frame recombination in the middle repeat region of *cagY*, which encodes an essential T4SS protein, though single nucleotide polymorphisms (SNPs) in *cagY* or in other genes that are essential for T4SS function may also occur (13). While we know how T4SS function is lost during experimental infection—or even gained (12)—we do not know why. The T4SS is typically functional during chronic infection in humans (38, 39) and in rhesus monkeys (26), unless essential genes are absent or present as pseudogenes. Yet, the extraordinary repetitive genetic structure of *cagY* and the demonstration that recombination in these repeat regions can alter T4SS function strongly suggest that there must be conditions under which T4SS-dependent inflammation is advantageous for the bacterium and others under which it is not. We know from studies with genetically modified mice that *cagY* recombination does not occur in the absence of CD4 T cells expressing IFN-γ, and so loss of T4SS function is immune sensitive (16). But these are not physiological experiments. Here, we sought to use the murine model to identify conditions relevant to human infection under which H. pylori modulates T4SS function.

Just as loss of T4SS function and *cagY* recombination do not occur in immunodeficient mice, they occur more commonly when the immune response is increased in IL-10 knockout mice, which have more severe gastritis and lower bacterial load when challenged with H. pylori (16). Since deletion of the *cag*PAI reduces inflammation and increases colonization (23, 24), loss of T4SS function may serve to maintain colonization in the face of increased inflammation, such as might occur during a severe, transient systemic infection. The notion that one infection can alter the outcome of another has been repeatedly demonstrated and is thought to occur via enhancement of nonspecific innate immunity (40–43). To test this hypothesis, we coinfected mice with H. pylori and *Salmonella*, predicting that, like in IL-10 knockout mice, we would find increased inflammation, lower bacterial load, and reduced T4SS function compared to that in mice infected with H. pylori alone. As expected, *Salmonella* coinfection decreased H. pylori colonization and was accompanied by increased gastritis as well as local and systemic levels of IFN-γ. The importance of IFN-γ was supported by the observation that its overexpression was itself sufficient to increase gastritis and reduce H. pylori colonization (Fig. 4). Similar to that in IL-10 knockout mice (16), H. pylori colonization in coinfected mice was inversely correlated with T4SS function, which we initially interpreted as consistent with immune escape leading to increased bacterial load.

However, in contrast to our prediction, T4SS function was increased in *Salmonella*-coinfected mice, and *cagY* recombination was reduced (Fig. 5). This suggested the possibility that *Salmonella* coinfection selected strains with a functional T4SS, which would be expected to be associated with more inflammation and therefore a lower bacterial load. Decreased H. pylori colonization during coinfection might then be a result of increased inflammation, not just from *Salmonella* infection but also from retention of T4SS function. Since the T4SS enables H. pylori to acquire iron from the host (28) and systemic infection causes iron sequestration (44), we asked whether iron deficiency might cause retention of T4SS function and explain why it is enhanced in *Salmonella* coinfection. While anemia *per se* had no effect, anemia induced by dietary iron restriction completely eliminated *cagY* recombination and loss of T4SS function (Fig. 6).

Together, these studies have identified two physiologically relevant variables that affect T4SS function in the murine model, *Salmonella* coinfection and especially dietary iron restriction, which completely eliminated loss of T4SS function. Both have epidemiologic parallels in humans, since H. pylori strains from individuals in developing countries, where iron deficiency and systemic infections are common, are also more often *cag*PAI^+^ than strains from developed countries. However, there are important caveats to our findings. For example, while the results of *Salmonella* coinfection prompted the iron restriction studies and both promote retention of T4SS function, it appears that they are not mechanistically linked, though we did not directly measure gastric iron levels. We currently do not have an explanation for why H. pylori in coinfected mice more often retains T4SS function. While iron deficiency maintains T4SS function and increases gastric IFN-γ, it does not affect colonization, which might be expected to decrease. It seems likely that the effects of *Salmonella* coinfection, and perhaps iron restriction, are pleomorphic and will require additional studies to understand their impact on T4SS function. One approach to separate the effects of *Salmonella* infection from the inflammation it causes might be to pharmacologically induce innate immunity with, for example, lipopolysaccharide (LPS) administration. Nonetheless, here we have identified two physiological variable factors relevant to human infection that affect T4SS function. The results have implications for our fundamental understanding of the *cag*PAI and also provide experimental tools that permit the study of T4SS function in the murine model.

## MATERIALS AND METHODS

### Ethics statement.

Experiments were carried out at the University of California, Davis, under protocols approved by the U.C. Davis Institutional Animal Care and Use Committee, which has been accredited by the Association for Assessment and Accreditation of Laboratory Animal Care. All animal experiments were performed in accordance with NIH guidelines, the Animal Welfare Act, and U.S. federal law.

### Bacterial culture.

H. pylori PMSS1 was cultured on brucella agar (BBL/Becton, Dickinson, Sparks, MD) supplemented with 5% heat-inactivated newborn calf serum (NCS; Invitrogen, Carlsbad, CA), ABPNV antibiotics (amphotericin B, 20 μg/ml; bacitracin, 200 μg/ml; polymyxin B, 3.3 μg/ml; nalidixic acid, 10.7 μg/ml; vancomycin, 100 μg/ml), and selective antibiotics (kanamycin, 25 μg/ml, or chloramphenicol, 5 μg/ml, where appropriate) (all antibiotics from Sigma). Prior to experimental mouse challenge, H. pylori was cultured overnight in brucella broth supplemented with 5% NCS and TVPA antibiotics (trimethoprim, 5 mg/liter; vancomycin, 10 mg/liter; polymyxin B, 2.5 IU/liter; amphotericin B, 2.5 mg/liter). Cultures were incubated at 37°C under microaerophilic conditions at 5% CO_2_ generated by an Anoxomat (Advanced Instruments, Norwood, MA). Construction of H. pylori PMSS1 Δ*cagE* and PMSS1 Δ*cagY* replaced with *cagY* from either PMSS1 (H. pylori PMSS1 *cagY*) or SS1 (H. pylori SS1 *cagY*) was previously described (16, 23). Salmonella enterica serovar Typhimurium BRD509 (strain SL1344 *aroA* mutant) was grown overnight in LB broth without shaking at 37°C prior to experimental challenge and enumerated by culture on MacConkey agar plates.

### Animals.

Female C57BL/6J WT and TCR β/δ^−/−^ mice were purchased from the Jackson Laboratory (Sacramento, CA). A male mouse with an extra copy of the mouse IFN-γ gene under the control of the H/K ATPase β promoter (H/K-IFN-γ line 944 mice) was provided by Andrzej Dlugosz (45). These tgIFN-γ mice were bred with WT and TCR^−/−^ mice to obtain heterozygous H/K-IFN-γ mice and H/K-IFN-γ^+/−^ TCR^−/−^ mice as well as littermate controls without the H/K-IFN-γ gene. Heterozygous erythropoietin knockout mice obtained from Nicolas Voituron (46) were bred in-house to obtain EPO^−/−^ mice and littermate controls. Mice were housed in microisolator cages and provided with irradiated food and autoclaved water *ad libitum*. Iron deficiency was induced by providing mice with food containing traces of iron in the range of 2 to 6 ppm (TD 10210; Envigo Teklad Diets, Madison, WI) starting from 3 weeks of age. Control mice were given an equivalent food but with 200 ppm iron (TD 150282; Envigo Teklad Diets). Food intake for each cage of 4 mice was monitored, and iron-deficient and -replete mice were weighed before and after H. pylori infection.

### H. pylori and *Salmonella* challenge.

At 8 to 9 weeks of age, mice were challenged with 1 × 10^9^ CFU of H. pylori suspended in 0.25 ml of brucella broth administered by oral gavage with a ball-end feeding needle. For coinfection experiments, mice were infected with *Salmonella* 1 week after H. pylori challenge by intravenous (i.v.) injection in the lateral tail vein with 5 × 10^5^ CFU of bacteria diluted in 0.2 ml phosphate-buffered saline (PBS). Bacterial concentrations were estimated by optical density at 600 nm and confirmed by plating serial dilutions. At the endpoint, mice were euthanized with an overdose of pentobarbital sodium injection (50 mg/ml intraperitoneally [i.p.]). Blood was collected in microcapillary tubes for hematocrit measurement and into serum separator tubes (BD Microtainer). The forestomach was removed, and the glandular stomach was cut longitudinally along the lesser curvature. Half of the stomach was placed in 300 μl brucella broth, weighed, ground with a sterile glass rod until the mucosal cells were homogenized, and then plated in serial dilution to determine CFU per gram. The limit of detection (LOD) for H. pylori was 20 CFU/g. *Salmonella* CFU were counted from homogenized stomach (LOD = 7.5 CFU/organ) or spleen (LOD = 24 CFU/organ). Absence of H. pylori or *Salmonella* colonies was represented as 0 CFU.

### Histology.

A quarter of the stomach was fixed in 10% formalin, sectioned, and stained with hematoxylin and eosin. The percentage of fields containing neutrophil infiltration (polymorphonuclear leukocytes), gastritis (mononuclear cells), and metaplasia was identified by a veterinary pathologist blinded to experimental condition, using a scoring system previously validated in mice (47). The results for the three histological criteria were averaged and defined as percent gastric inflammation.

### Gene expression and iron analysis.

Serum IFN-γ levels were analyzed with the mouse IFN-γ uncoated ELISA kit according to the manufacturer’s protocol (Invitrogen). Murine gene expression of IFN-γ, hepcidin, and lipocalin-2 was analyzed from gastric tissue homogenized in TRIzol reagent (Ambion). Bacterial expression of *fur* was analyzed from sweeps isolated from gastric tissue and cocultured for 20 h with AGS cells as described below. RNA was purified by phenol-chloroform phase separation as described by the manufacturer or by the Direct-zol RNA miniprep kit (Zymogen). RNA was transcribed to cDNA by Superscript III (Qiagen) and added to quantitative PCR (qPCR) mixtures with TB green premix Ex *Taq* (TaKaRa) using primers shown in Table S1 in the supplemental material. Gene expression was normalized to murine glyceraldehyde-3-phosphate dehydrogenase (GAPDH) or bacterial 16S rRNA expression. Amplification was performed using a QuantStudio 6 Flex real-time PCR system (Applied Biosystems). Total serum iron was analyzed on a chemistry analyzer by the Comparative Pathology Laboratory at UC Davis.

10.1128/mBio.03147-20.4 TABLE S1 Primers used for real-time PCR. Download Table S1, DOCX file, 0.1 MB. Copyright © 2020 Skoog et al. 2020 Skoog et al. This content is distributed under the terms of the Creative Commons Attribution 4.0 International license.

### IL-8 ELISA.

IL-8 was measured as described previously (48). Briefly, human AGS gastric adenocarcinoma cells (ATCC, Manassas, VA) were grown in RPMI 1640 medium supplemented with 10% fetal bovine serum, 100 units/ml penicillin, and 100 μg/ml streptomycin at 5% CO_2_ and 37°C. The cells were seeded in six-well plates at a density of approximately 5 × 10^4^ cells/cm^2^ with 1.8 ml antibiotic-free RPMI medium-10% fetal bovine serum, incubated overnight, and then cocultured with H. pylori sweeps from an individual mouse diluted in 200 μl brucella broth at a multiplicity of infection (MOI) of 100:1. Supernatants were harvested after 20 to 22 h of culture (37°C, 5% CO2) and diluted 1:8 prior to IL-8 assay by ELISA (Invitrogen) performed according to the manufacturer’s protocol. WT H. pylori PMSS1 and the isogenic *cagY* deletion mutant were included on every plate as positive and negative controls, respectively. IL-8 expression was normalized to that for WT PMSS1.

### *cagY* PCR restriction fragment length polymorphism.

*cagY* genotyping was performed on isolated single colonies by PCR-restriction fragment length polymorphism (RFLP) essentially as previously described (12). *cagY* was amplified with Herculase II fusion DNA polymerase (Agilent Technologies), digested with DdeI and BfuCI or Sau3AI restriction enzymes (New England BioLabs, Ipswich, MA), visualized by agarose gel electrophoresis, and compared to that for WT H. pylori PMSS1. For convenience, *cagY* recombination was defined as a change in PCR-RFLP pattern compared to that for the WT, though we did not formally measure recombination and changes could occur by other mechanisms.

### Statistics.

All statistical analyses were performed using GraphPad Prism 7.00 (GraphPad Software, San Diego, CA). Multiple groups were compared using analysis of variance (ANOVA) with Tukey’s or Bonferroni’s *post hoc* tests. Differences in H. pylori colonization (CFU/g) and IL-8 expression between two groups were analyzed using the Mann-Whitney test. Proportions of samples with changed *cagY* were compared between groups using the Fisher’s exact test. Correlation between IL-8 expression and H. pylori CFU was analyzed by linear regression. Data are reported as means ± standard errors of the means (SEMs), and a *P* value of <0.05 was considered statistically significant.
