# Supplementary figures and images for: Maintenance of Type IV Secretion Function During Helicobacter pylori Infection in Mice
Source: mBio. 2020 Dec 22;11(6):e03147-20. doi: 10.1128/mBio.03147-20 (PMC8534286; doi:10.1128/mBio.03147-20)

FIGURE S1

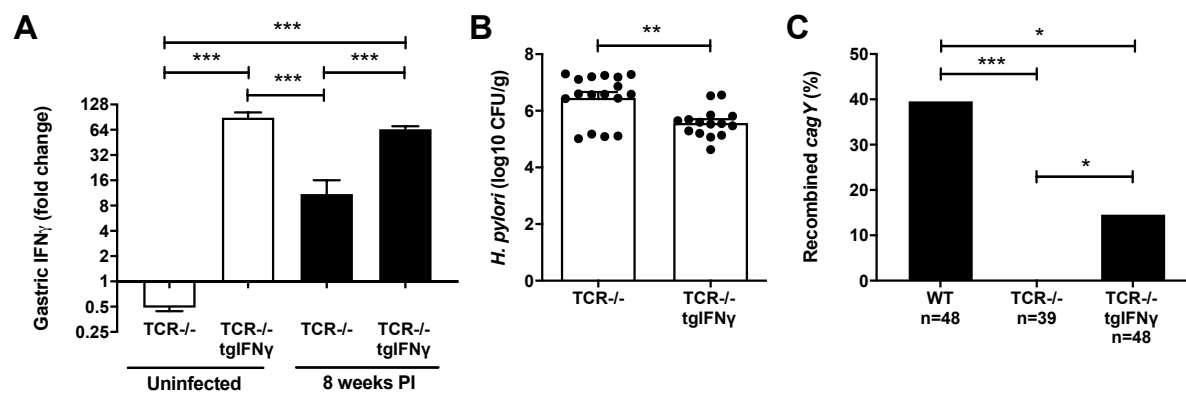

Supplement: FIG S1 [file mbio03147-20-sf001.pdf]

FIGURE S2

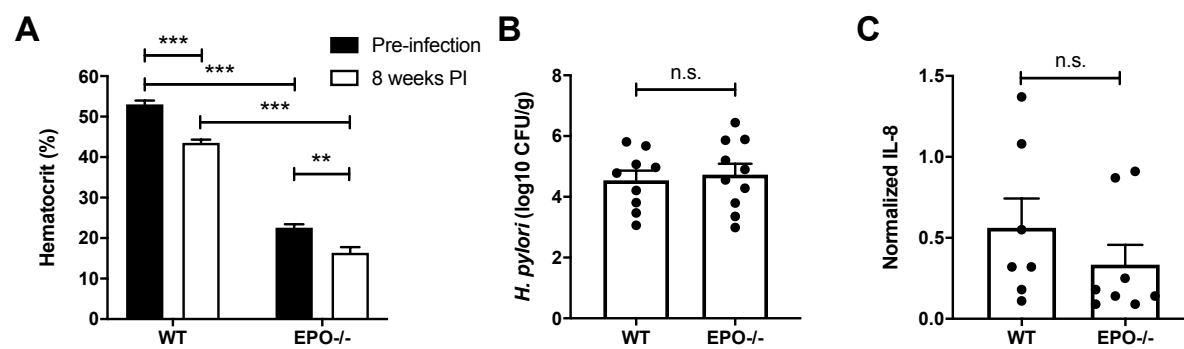

Supplement: FIG S2 [file mbio03147-20-sf002.pdf]

FIGURE S3

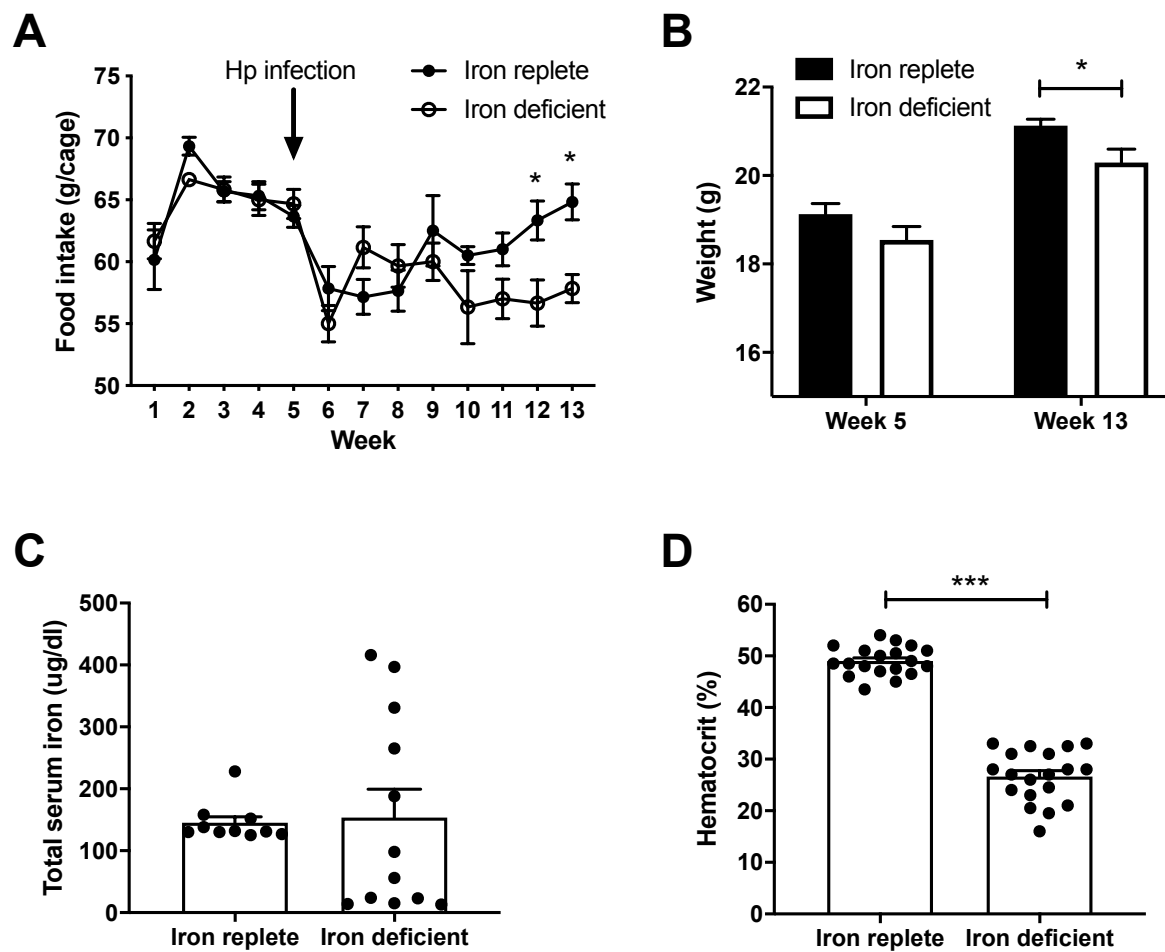

Supplement: FIG S3 [file mbio03147-20-sf003.pdf]
