# Supplementary material for: Maintenance of Type IV Secretion Function During Helicobacter pylori Infection in Mice
Source: mBio. 2020 Dec 22;11(6):e03147-20. doi: 10.1128/mBio.03147-20 (PMC8534286; doi:10.1128/mBio.03147-20)
Supplement: TABLE S1 [file mbio03147-20-st001.docx]

# TABLE

**Table S1. Primers used for real-time PCR**

| **Target** | **Forward 5’-3’** | **Reverse 5’-3’** | **Reference** |
| --- | --- | --- | --- |
| IFNγ | GAGGTCAACAACCCACAGG | CCGAATCAGCAGCGACTCCT | (45) |
| Hepcidin | AGAGCTGCAGCCTTTGCAC | GAGGTCAGGATGTGGCTCTA | (49) |
| Lipocalin2 | CTGAATGGGTGGTGAGTGTG | GCTCTCTGGCAACAGGAAAG | (49) |
| GAPDH | GATGACATCAAGAAGGTGGTGAA | AAGAGTGGGAGTTGCTGTTGAA |  |
| fur | GAAGAAGTGGTGAGCGTTTTG | CCTTTTGGCGGATAGAATGC | (50) |
| 16S rRNA | GGAGTACGGTCGCAAGATTAAA | CTAGCGGATTCTCTCAATGTCAA |  |
